# Factors associated with preoperative and postoperative epileptic seizure in patients with cerebral ganglioglioma

**DOI:** 10.12669/pjms.302.4362

**Published:** 2014

**Authors:** Cheng Huang, He Li, Mingwan Chen, Yang Si, Ding Lei

**Affiliations:** 1 Cheng Huang, Department of Neurology, West China Hospital of Sichuan University, China.; 2 He Li, West China School of Medicine, Sichuan University, China.; 3 Mingwan Chen, West China School of Medicine, Sichuan University, China.; 4 Yang Si, Department of Neurology, West China Hospital of Sichuan University, China.; 5 Ding Lei, Department of Neurosurgery, West China Hospital of Sichuan University, China.

**Keywords:** Cerebral ganlioglioma, Epilepsy, Neurosurgery

## Abstract

***Objective:*** To explore the factors associated with preoperative epileptic seizure and surgical outcome in patients with cerebral gangliolioma (GG).

***Methods:*** A total of 31 consecutive patients with pathologically confirmed ganglioglioma and surgically treated from January 2003 to June 2011 in West China Hospital of Sichuan University were retrospectively reviewed. Clinical data, surgical procedure and follow-up information were collected and analyzed.

***Results:*** Nineteen patients presented with epileptic seizure, of which 63.2% were males. The mean age at epilepsy surgery and mean seizure duration were 25.6 years and 2.3 years respectively. Factors associated with preoperative epileptic seizure were supratentorial lesion and temporal lobe involvement (p=0.016 and 0.008). Intraoperative electrocorticography (ECoG) was applied in 8 out of 19 epilepsy patients. Eighteen achieved total tumor excision. After a mean follow up of 2.8 (1.3-6.3) years, 11 (68.8%, 11/16) achieved seizure free (Engel class I). Early surgery (seizure duration <3 years) was a significant predictor of favorable seizure outcome (*p*=0.013). None of the factors including seizure type, tumor location, neuroimaging characteristics and application of intraoperative ECoG or surgical strategy were found to be significantly associated with postoperative seizure outcome. Postoperative combination of AEDs was unnecessary for seizure control.

***Conclusions:*** Ganglioglioma with temporal lobe involvement usually associated with intractable epilepsy. Early surgical resection is strongly suggested to achieve favorable outcome. Intraoperative ECoG is not inevitable and simple lesionectomy is sufficient for satisfactory seizure control. Early accurate diagnosis of ganglioglioma should be established on comprehensive consideration and plays an important role in dealing with these patients.

## INTRODUCTION

Ganglioglioma (GG) is a type of rare neuroepithelial tumor of the central nervous system (CNS) and accounts for about 0.4% to 2% of all CNS tumors.^1^ It contains ganglion cells and neoplastic glial (astrocytic or oligodendroglial) elements pathologically.^2^ GG is considered as a common cause of tumor-related refractory epilepsy besides other low-grade gliomas, especially in patients younger than 30 years.^3^ It is reported that GGs accounts for about 40% of all epileptogenic tumors and temporal lobe gangliogliomas are almost always found to show epileptogenicity.^4^^,^^5^ Given the low incidence of GG, risk factors for preoperative epileptic seizure and postoperative outcome are still controversial. The purpose of our study was to further explore the characteristics of GG and factors associated with clinical presentation and surgical outcome. 

## METHODS

***Patient selection: ***Thirty-one patients with pathologically confirmed ganglioglioma and surgically treated at West China Hospital of Sichuan University during the period from January 2003 to June 2011 were retrospectively studied. All patients were first operated in our institution without tumor resection before.

***Clinical data collection: ***Clinical data of gender, age, disease course, neurological manifestation at admission, diagnostic procedures, neuroimaging characteristics, size and surgical procedures were collected. Family history of epileptic seizure, pre- and peri- and postnatal factors, history of tobacco and alcohol use was collected. Each patient obtained at least one EEG and brain magnetic resonance imaging (MRI) scan before surgery. MRI images were reviewed to describe the site, size, cystic changes as well as contrast enhancement, calcification and mass effect of the tumor. We assigned patients with preoperative epilepsy into epilepsy group (E-group) and those with headache or FND into non-epilepsy group (NE-group). 

***Surgical procedure: ***All patients underwent surgical procedure by senior neurosurgeons in our hospital. The surgical strategy and extent of resection was determined by the location and characteristics of the tumor. We grouped extent of resection into two groups according to the surgical records: gross total excision and subtotal/partial excision.

***Follow-up: ***Follow-up information was obtained by out-patient and telephone interviews. The surgical outcome of patients with epilepsy was classified according to the Engel’s classification into class I, completely seizure free for at least 2 years, auras only, or convulsions with drug withdrawal only; class II, rare seizure (≤2 seizures per year); class III, worthwhile improvement; class IV, no significant improvement or worse. 

***Statistic analysis: ***Factors associated with preoperative epileptic seizure and surgical outcome were estimated by using Student’s t test (two-tailed) for quantitative variables and Fisher’s exact test (two-tailed) for non-parametric factors. Significance was defined as P<0.05. The analysis of risk factors for epilepsy in patients with GGs was estimated by calculating odds ratios (OR), with 95% confidence intervals (95% CI). The significance level was set at 0.05. Variables which would potentially predict increasing risk for epilepsy were then evaluated by logistic regression model. 

## RESULTS

***Factors associated with preoperative epileptic seizure:***

***Patient information: ***The study consisted of 19 (61.3%) patients with preoperative epilepsy and 12 (38.7%) without (Table-I). In epilepsy group, 2 presented with simple partial seizure, 8 complex partial seizure and 9 secondary generalized seizure. Nine patients (47.4%) were drug resistant. In non-epilepsy group, 9 (0.75%) had headache and the others had FND at admission. There were 12 male patients in E-group while only 2 in NE-group (*p*=0.024). No significant difference of mean age at onset, disease course or age at surgery was found between two groups.

***Tumor characteristics: ***Each patient in this study had only one lesion. As a total, 11 tumors were confined in the temporal lobe, 7 in frontal lobe, 3 in frontoparietal lobe, 4 in deep brain area, 3 in cerebellum, 1 in brain stem, 1 with frontal lobe and deep brain area involved and 1 with temporal lobe and deep brain area involved. There were 9 (29.3%) tumors with cystic changes, 7 (22.6%) with contrast enhancement, 7 (22.6%) with peripheral edema, 8 (25.8%) with mass effect and 5 (16.1%) with calcification. Factors having significant association with preoperative epileptic seizure were supratentorial location (*p*=0.016) and temporal lobe involvement (*p*=0.008). However, no significant difference of these factors was found between two groups. Size of tumor and pathological grading results had no correlation with preoperative seizure (*p*=0.433 and 0.142).

**Table-I:** Basic characteristics and preoperative factors for epileptic seizure of 31 patients with ganglioglioma (fisher’s).

*Factors*	*Epilepsy group*	*Non-epilepsy group*	*p value*
Number	19	12	-
Gender (male/female)	12/7	2/10	0.024
Mean age at symptom onset (y ± SD)	23.3 ± 14.3	24.6 ± 18.0	0.823
Mean age at surgery (y ± SD)	25.6 ± 13.5	26.1 ± 17.4	0.928
Supratentorial vs. infratentorial	19/0	8/4	0.016
Multiple lobe vs. single lobe	3/16	2/10	1.000
Temporal lobe (yes/no)	11/8	1/11	0.008
Cerebellum (yes/no)	0/19	3/9	0.049
Cystic changes (yes/no)	7/12	2/10	0.418
Contrast enhancement (yes/no)	3/16	4/8	0.704
Peripheral edema (yes/no)	4/15	3/9	1.000
Mass effect (yes/no)	3/16	5/7	0.253
Calcification of the tumor (yes/no)	3/16	2/10	1.000
Size (>3cm or not)	13/6	10/2	0.433
WHO grade of tumor (I,II vs. III)	19/0	10/2	0.142

***Seizure outcome: ***Followed-up information of patients with epilepsy was collected. Three patients (15.8%, 3/19) lost follow-up. After a mean follow-up of 34.1 months (range from 15 to 75 months), 11 patients achieved seizure free (Engel class I) and of which 9 accepted monotherapy and 2 accepted duotherapy of AEDs postoperative. Only 4 had stopped antiepileptic drug (AED) intake at final follow-up.

***Factors associated with postoperative seizure outcome:*** Gender, mean age at seizure onset and at surgery had no significant effect on surgical outcome (*p*=0.588, 0.827 and 0.621) (Table-II). Early surgery (seizure duration less than 3 years) predicted better seizure outcome (*p*=0.013). Preoperative seizure frequency and seizure type were similar (*p*=0.951 and 0.245). Tumor location did not affect surgical outcome. Other neuroimaging characteristics were not correlated with seizure outcome. Surgical resection type were similar in two groups (*p*=1.000). There was only one patient in seizure-free group had undergone subtotal excision considering preserve functional brain area. The use of intraoperative ECoG did not predict better outcome (*p*=0.282). Monotherapy or combination of AEDs postoperatively showed no significant diffenrence on seizure control (*p*=0.547). Other factors including tobacco use, alcohol intake and family history of epilepsy as well as per, peri- or post-natal factors, febrile seizure and brain trauma or surgery history were found no significant association with seizure outcome (*p*=0.547, 1.000, 1.000, 0.214, 1.000 and 0.083 respectively) (not shown in Table-II).

**Table-II:** Factors for postoperative seizure outcome of 16 patients with gangliolioma (fisher’s).

*Factors*	*Seizure-free (Engel I, n=11 )*	*Non-seizure-free (Engel II/ III, n=5 )*	*p value*
Gender: male vs. female	6/5	4/1	0.588
Mean age at seizure onset (y ± SD)	22.4 ± 15.6	20.6 ± 12.1	0.827
Mean age at surgery (y ± SD)	23.2 ± 15.8	27.0 ± 8.2	0.621
Disease course <3 years	10/1	1/4	0.013
Seizure frequency (/m ± SD)	1.96 ± 1.47	2.00 ± 1.00	0.951
Seizure type: SPS/CPS vs. SGS	9/2	2/3	0.245
Multiple lobe vs. single lobe	3/8	0/5	0.509
Temporal lobe (yes/no)	6/5	3/2	1.000
Cystic changes (yes/no)	6/5	0/5	0.093
Contrast enhancement (yes/no)	3/8	2/3	1.000
Peripheral edema (yes/no)	3/8	0/5	0.509
Mass effect (yes/no)	2/9	1/4	1.000
Calcification of the tumor (yes/no)	2/9	1/4	1.000
Size >3cm (yes/no)	7/4	3/2	1.000
Intraoperative ECoG (yes/no)	4/7	4/1	0.282
Total vs. subtotal/partial excision	10/1	5/0	1.000
Monotherapy/duotherapy	9/2	3/2	0.547

## DISCUSSION

Ganglioglioma is a type of rare neuroepithelial tumor of the central nervous system which has been increasingly recognized as a common neoplastic cause of medically refractory epilepsy which accounts for about 40% of all epileptogenic tumors and temporal lobe GGs almost always show epileptogenicity.^4^^,^^5^ However, according to our clinical experience, this kind of tumor has not been well recognized in the developing countries until recent years. Gangliogliomas were usually preoperative misdiagnosed as vascular lesions, astrocytomas, gliomas, maningiomas, or even inflammation lesions sometimes.^6^^,^^7^ In this study, we discussed about the clinical and neuroimaging characteristics of GG and the factors associated with preoperative epileptic seizure and surgical outcome. 

In our study, 31 patients with GG underwent surgery and the most common presenting symptom was epileptic seizure (61.3%), of which about 50% were drug resistant. We found GG have a slight predilection for the male gender with a proportion of 63.2%. This result is in accordance with that in a systematic review conducted by Isaac Yang which is around 56%. Temporal lobe involvement was in tight association with preoperative epileptic seizure in our study and it was supported by several other research.^8^^,^^9^ Our inability to find most of the variables, especially tumor characteristics with preoperative epileptic seizure may due to limited number of sample size.

Although surgical resection is regarded as a curative treatment and important method of seizure control for GG, factors associated with surgical outcome remains controversial. A number of studies showed patients with shorter epileptic duration and younger age at surgery achieved better outcome.^9^^-^^12^ In our study, 90.9% epileptic patients with disease course less than 3 years achieved seizure-free and no one suffered tumor recurrence or progression. Completed tumor resection was considered as an important prognostic factor for favorable seizure outcome in several studies.^4^^,^^11^^,^^13^ However, a number of researches found it was not inevitable.^14^^-^^16^ In this study, we found no significant difference of seizure outcome between incomplete and complete resection. This may need further investigation due to limited number of patients with incomplete surgical resection.

Intraoperative ECoG is a commonly used technique in epilepsy surgery to help identifying the epileptogenic zone. Several studies recommended surgical resection should include both tumor and ECoG active area to achieve favorable seizure outcome.^4^^,^^17^ However, due to its own limitations such as short duration, lack of ictal records and anesthesia disturbance, some researchers also found satisfactory seizure control by lesionectomy alone without intraoperative ECoG.^14^ In our study, the seizure-free rate in patients with (n=8) and without (n=8) introperative ECoG was 50% and 87.5%. This difference reached no statistical significance and it is even slightly higher in patients without intraoperative ECoG. Therefore, considering the limited benefits and results from other studies, we suggest that intraoperative ECoG is not essential in epilepsy surgery for ganglioglioma especially in low-income population in developing countires.

Continued AED treatment after surgery is crucial as surgery alone is unlikely to completely control seizure due to possible persistence of an epileptogenic network.^18^ However, experts hold different views on choice of monotherapy or combination of AEDs.^19^^,^^20^ In our study, 75% patients received monotherapy achieved seizure free at final follow-up and duotherapy of polytherapy after successful surgery seemed unnecessary. Although several studies insist on combination of AEDs, we suggest monotherapy is an effective therapeutic strategy when dealing with patients diagnosed as ganglioglioma.

In our study, we failed to find association between other factors and surgical outcome, including tumor characteristics, family history of epilepsy, tobacco or alcohol intake, pre-, peri- or post-natal factors, febrile seizure and brain trauma or surgery history. The underlying mechanism of epileptogenicity of ganglioglioma may need further basic studies to find out more accurate prognostic factors.

The primary limitations of this study were its retrospective nature and limited sample size. However, as relative low incidence of ganglioglioma and difficulty in diagnosis in developing regions, it is sometimes reasonable to explore related factors through retrospective study to see whether a study with larger sample size is worthwhile. 

## CONCLUSIONS

In conclusion, ganglioglioma with temporal lobe involvement usually associated with intractable epilepsy. Early surgical resection is strongly suggested to achieve favorable outcome. Intraoperative ECoG is not inevitable and simple lesionectomy is sufficient for satisfactory seizure control. Postoperative combination of AEDs was unnecessary for seizure control. In developing countries, ganglioglioma were not well known until recent years and were usually misdiagnosed presurgically. Early accurate diagnosis of ganglioglioma should be established on comprehensive consideration and plays an important role in dealing with these patients.
